# Proliferation Marker Ki67 as a Stratification Index of Adjuvant Chemotherapy for Resectable Mucosal Melanoma

**DOI:** 10.3389/fonc.2022.895672

**Published:** 2022-06-30

**Authors:** Lirui Tang, Xiaoting Wei, Caili Li, Jie Dai, Xue Bai, Lili Mao, Zhihong Chi, Chuanliang Cui, Bin Lian, Bixia Tang, Yu Du, Xuan Wang, Yumei Lai, Xinan Sheng, Xieqiao Yan, Siming Li, Li Zhou, Yan Kong, Zhongwu Li, Lu Si, Jun Guo

**Affiliations:** ^1^ Key Laboratory of Carcinogenesis and Translational Research (Ministry of Education, Beijing), Department of Melanoma and Sarcoma, Peking University Cancer Hospital and Institute, Beijing, China; ^2^ Key Laboratory of Carcinogenesis and Translational Research (Ministry of Education, Beijing), Department of Pathology, Peking University Cancer Hospital and Institute, Beijing, China; ^3^ Key Laboratory of Carcinogenesis and Translational Research (Ministry of Education, Beijing), Department of Genitourinary Cancers, Peking University Cancer Hospital and Institute, Beijing, China

**Keywords:** Ki67, adjuvant chemotherapy, HDI, relapse-free survival (RFS), mucosal melanoma (MM), melanoma-specific survival, stratification index

## Abstract

**Background:**

Adjuvant chemotherapy has been shown to produce a favorable prognosis for patients with resectable mucosal melanoma (MM), resulting in the need for stratification to optimally select patients to benefit from adjuvant therapy. This study analyzed Ki67 as a potential stratification index for adjuvant chemotherapy in resectable MM.

**Methods:**

Patients with resected MM who received subsequent adjuvant therapy in Beijing Cancer Hospital between 2010 and 2018 were retrospectively enrolled and analyzed. Relapse-free survival (RFS) and melanoma-specific survival (MSS) curves were used to perform the survival comparisons across different subgroups.

**Results:**

From Jan 2010 to Dec 2018, 1106 MM patients were screened from a database of 4706 patients and 175 of these patients were finally enrolled. A total of 100 patients received temozolomide (TMZ)-based adjuvant chemotherapy and 75 patients received high-dose interferon-α2b (HDI) adjuvant therapy. Compared with HDI, patients who received TMZ-based adjuvant chemotherapy had significantly superior RFS (21.0 vs. 9.6 months, P = 0.002). For patients with low Ki67 expression (<30%), the two regimens showed no significant difference for impact on RFS (33.9 vs. 22.7 months, P = 0.329). However, for patients with high Ki67 expression (≥30%), TMZ-based adjuvant chemotherapy achieved favorable RFS compared with HDI (18.0 vs. 6.7 months, P < 0.001) and tended to improve MSS compared to HDI (41.4 vs. 25.1 months, P = 0.067).

**Conclusion:**

Compared with HDI, adjuvant chemotherapy may be more relevant for patients with Ki67 ≥ 30%. Ki67 may serve as a potential index to distinguish populations benefiting from adjuvant chemotherapy in resectable MM, and may provide a basis for stratification in the selection of adjuvant regimens.

## Introduction

Primary mucosal melanoma (MM), which accounts for approximately 1.3% of all melanomas occurring in Caucasian populations, is a rare but fatal malignancy arising from melanocytes (1–3). Conversely, in the Chinese population, mucosal melanoma represents a much larger proportion of melanoma cases, with estimates in the range of 22-25% of all melanomas (4).

MM displays distinctive biological behavior that makes it more prone to invasion by vessels. In this way, MM is consequently more prone to recurrence or distant metastasis and is often diagnosed at an advanced stage. For metastatic MM, no standard treatment has been established, resulting in poor survival compared to cutaneous melanoma (5). As for patients with MM, the primary treatment is surgical resection when feasible, but that even in patients with resectable disease, there is a significant risk of recurrence, and therefore adjuvant chemotherapy may provide a favorable prognosis. A phase II randomized trial comparing high-dose interferon (IFN)-α2b (HDI) with temozolomide (TMZ)-based adjuvant therapy for resected MM suggested that TMZ-based adjuvant chemotherapy may have a more favorable prognosis than HDI (6). Subsequent to the phase II results, Lian and colleagues presented the results of a randomized phase III study in abstract form, in which they demonstrated that TMZ-based adjuvant chemotherapy significantly reduced the risk of relapse and metastasis in patients with resected MM compared with adjuvant HDI. Importantly, these studies predated the use of immunotherapy in MM (7). In recent years, a series of clinical trials have shown that adjuvant anti-programmed cell death protein 1 (PD-1)-based therapy was effective for patients with resectable cutaneous melanoma. The KEYNOTE-054 and the CheckMate-238 studies demonstrated that as adjuvant therapy for patients with resected high-risk melanoma, pembrolizumab and nivolumab can significantly prolong relapse-free survival (RFS) compared with placebo or ipilimumab. However, both of these studies focused on cutaneous melanoma and involved a limited number of patients with MM (8, 9). While a phase randomized trial comparing adjuvant anti-PD-1 antibody (Toripalimab) versus HDI in resected MM suggested that the two regimens were not significantly different in their impact upon RFS (10). Greater clarity on the role of chemotherapy in the adjuvant treatment of patients with resectable MM has been obtained in these trials, but a stratification index is still needed to distinguish the optimal population which can benefit from adjuvant chemotherapy.

As a hallmark of cancer, the continued proliferation of tumor cells leads to rapid tumor invasion and metastasis. Ki67 is an important indicator of the cell proliferation rate for evaluating tumor malignancy and has been used to predict prognoses for various malignant tumor types (11). Recent studies using 30% as the cut-off Ki67 value for stratified analysis have likewise determined that a high Ki67 score (30%) is a potential prognostic marker and predictor of neoadjuvant or adjuvant chemotherapy efficacy in breast cancer and other malignant diseases (12–16). More specifically, a series of studies have indicated that the Ki67 level appears to be a strong and robust predictor of metastasis as well as an indicator of poor prognosis in melanoma (17–20). However, to the best of our knowledge, there is still a lack of evidence to support its role in melanoma treatment. Hence, the present study aimed to explore correlations between Ki67 levels and the efficacy of adjuvant chemotherapy in MM as well as to provide an accurate basis for selecting the optimal population for adjuvant therapy.

## Methods

### Patients

**Figure 1:**
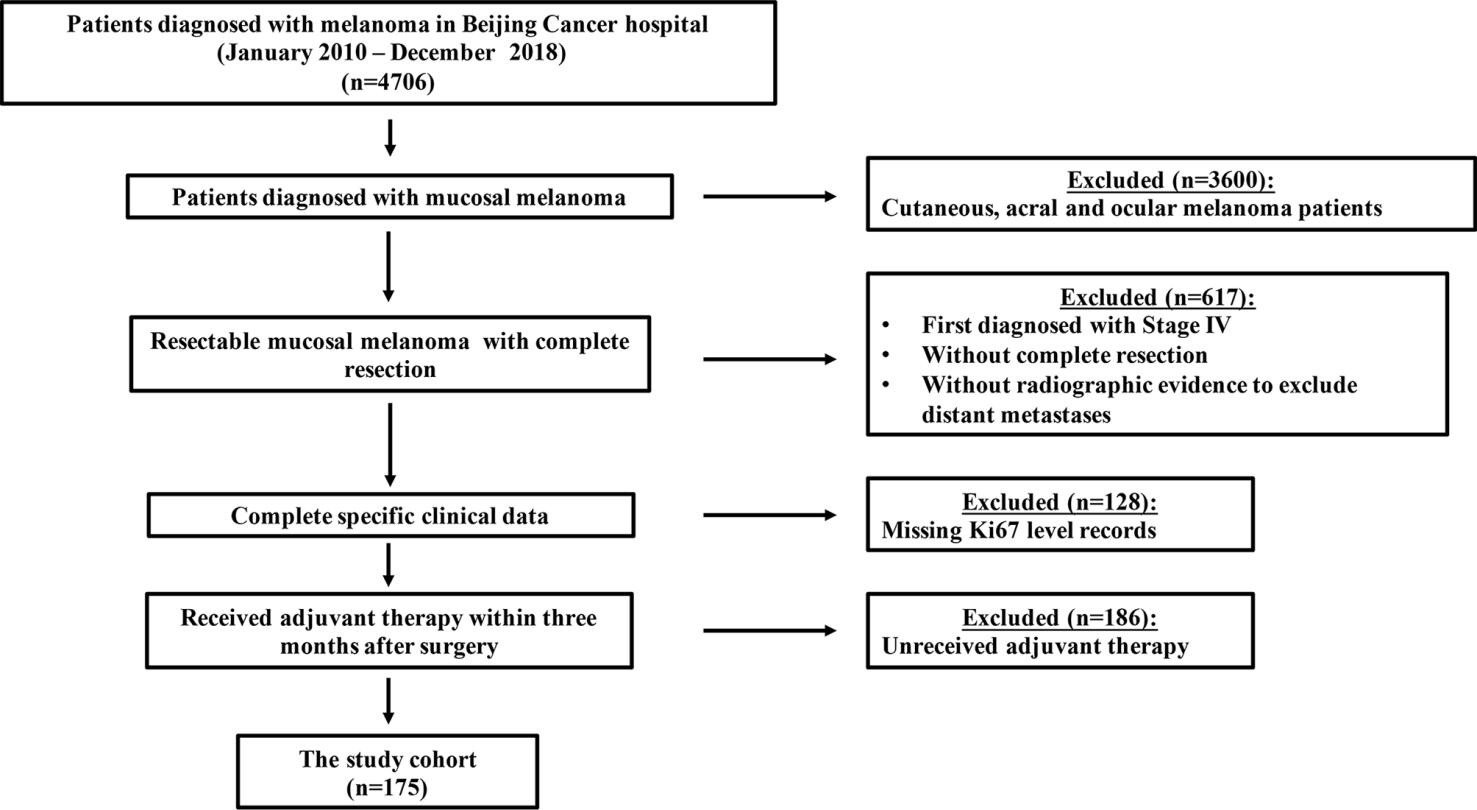
Flowchart of inclusion and exclusion criteria for this study. A total of 1106 MM patients were screened from a database of 4406 patients, and 175 patients were finally enrolled.

This was a retrospective study. Eligibility criteria for enrollment were 1. Pathological diagnosis of MM which excluded distant metastases by lymph node ultrasonography, anorectal and genitourinary tract ultrasonography, single-photon emission computed tomography (CT), whole-body spiral CT or positron emission tomography-CT (PET-CT) in Beijing Cancer Hospital; 2. Diagnosis data ranging between Jan. 1, 2010, and Dec. 31, 2018; 3. Complete resection was received; 4. Ki67 was identified by immunohistochemical (IHC) staining, and Ki67 level records were documented; and 5. TMZ-based adjuvant chemotherapy (per os 200 mg/m2/d TMZ on days 1 to 5 plus intravenous injection (i.v.) 75 mg/m2 cisplatin divided into 3 days repeated every 3 weeks for a total of six cycles), or HDI adjuvant therapy (i.v. 15 × 106 U/m2/d IFN-α2b on days 1 to 5 of each week for 4 weeks, followed by subcutaneous injection (s.c.). three weekly doses of 9 × 106 U IFN-α2b) was received within 3 months after surgery. We excluded patients with a diagnostic date after 2018 due to inadequate follow-up time (**Figure 1**). Clinical and pathological data from patients with resected MM who received adjuvant therapy were collected for analysis. Data for collection included age, sex (male vs female), primary site (nasal cavity vs oral vs urinary system vs reproductive system vs esophageal vs rectum), Ki67 level, initial lymphatic metastasis (yes vs no), adjuvant therapy regimen, lactate dehydrogenase (LDH) level, and gene mutational status (*BRAF*, *c-KIT*, *NRAS*: mutated vs wild-type vs unknown). This study was approved by the Medical Ethics Committee of the Beijing Cancer Hospital and Institute and was conducted according to the Declaration of Helsinki Principles. The studies involving human participants were reviewed and approved by Institutional Review Boards of Peking University Cancer Hospital. The patients/participants provided their written informed consent to participate in this study.

### Immunohistochemistry

Formalin-fixed, paraffin-embedded patient tissue samples were cut into 5-mm sections. Ki67 was identified by IHC staining by automated immunostainer (Link 48; Dako, Carpinteria, CA, USA), and was detected in the nuclei of tumor cells. The IHC marker used was monoclonal mouse anti-human Ki67 (clone MIB-1; Dako). IHC results were evaluated independently by two different pathologists who had no background knowledge related to the subject. If their opinions differed, a third pathologist reviewed the results.

### Clinical Outcome Assessment

RFS was defined as the time from the start of adjuvant treatment to the date of recurrence or death, whichever came first. MSS was defined as the time from the initial diagnosis date to the melanoma-specified death date. If the patient was lost to follow up or was still alive, then survival was measured at the last date that the patient was known to be alive. MSS was measured at the death date if the patient died from other reasons not related to melanoma.

### Statistical Analyses

According to a conventional selection of cut-off values, we defined patients with Ki67 expression above 30% positive tumor cells as the Ki67 high group; otherwise, they were considered in the Ki67 low group (12–16). The Kaplan-Meier method was used to analyze the RFS and MSS curves, and log-rank tests were used to perform the survival comparisons across different subgroups. Hazard ratios (HRs) of different Ki67 levels and adjuvant treatments (TMZ-based vs HDI) for RFS and MSS in all analyses were calculated by multivariate Cox proportional hazards models adjusting for age, sex, primary site, initial lymphatic metastasis, lactate dehydrogenase level, and gene mutational status.

All analyses were performed using Statistical Analysis System (SAS) software version 9.4 (SAS Institute, Inc., Cary, NC, USA), and a two-tailed P value < 0.05 was considered statistically significant.

## Results

### Patient Characteristics

Between January 2010 and December 2018, a total of 1106 MM patients were screened from a database of 4706 patients, and 175 patients were finally enrolled in this study. Baseline demographics are summarized in **Table 1**. The male to female ratio was 1:1.73 (64 vs. 111). The median age was 60.0 (range, 26.0–86.0) years. Ki67 < 30% accounted for 29.1% (51/175) of patients and Ki67 ≥ 30% accounted for 70.9% (124/175) of patients. In terms of adjuvant treatment options, 100 patients (57.1%) received TMZ-based adjuvant chemotherapy and 75 patients (42.9%) received HDI adjuvant therapy. The primary sites of resectable MM were nasal cavity and sinuses (36.6%), oral cavity (9.7%), anorectal(16.6%), genital tract (26.9%), urinary tract (3.4%) and esophagus (6.8%). Lymphatic metastasis was confirmed at the primary diagnosis in 31 patients (17.7%), and 6.3% (11/175) of patients had abnormal LDH levels. Hotspot mutational analysis was performed in 149 patients, and 5.1% (9/175) of patients were detected with the *BRAF* mutation, 4.6% (8/175) with the *c-KIT* mutation, and 9.1% (16/175) with the *NRAS* mutation.

**Table 1:** Baseline demographics, clinical characteristics and adjuvant therapy regimens of resectable MM patients.

Characteristic	No. % (n = 175)
**Age**	
Median	60.0
Range	26.0-86.0
**Age category**	
<65	116 (66.3)
>=65	59 (33.7)
**Sex**	
Female	111 (63.4)
Male	64 (36.6)
Primary Site*	
Nasal cavity and sinuses	64 (36.6)
Oral cavity	17 (9.7)
Anorectal	29 (16.6)
Genital tract*	47 (26.9)
Urinary tract	6 (3.4)
Esophagus	12 (6.8)
**Lymphatic metastasis**	
N	144 (82.3)
Y	31 (17.7)
**Mutation status**	
*BRAF*	9 (5.1)
*c-KIT*	8 (4.6)
*NRAS*	16 (9.1)
Wild type	116 (66.3)
Unknown	26 (14.9)
**LDH level**	
≤ULN	158 (90.3)
>ULN	11 (6.3)
Unknown	6 (3.4)
**Adjuvant therapy**	
TMZ-based Chemotherapy	100 (57.1)
High dose IFN-α2b	75 (42.9)

*Genital tract included the uterine cervix (6 cases)_and vulvovaginal primary lesions (41 cases).

LDH, lactate dehydrogenase; ULN, upper limit of normal; TMZ, temozolomide; IFN, interferon.

### Survival Profiles

**Figure 2:**
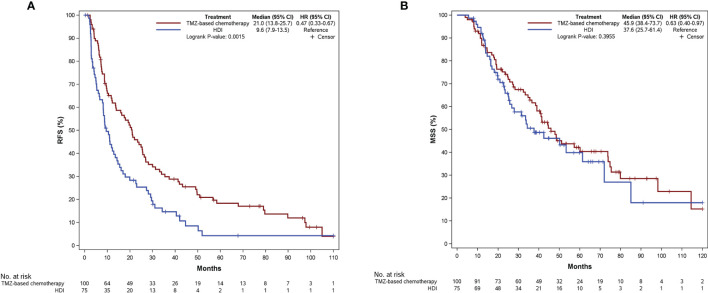
Kaplan-Meier curves of RFS **(A)** and MSS **(B)** according to patients receiving different adjuvant regimens. RFS, relapse-free survival; MSS, melanoma-specific survival; TMZ, temozolomide; HDI, high-dose interferon-a2b.

The last follow-up was December 1, 2021, with a median follow-up time of 59.4 months (95% confidence interval [CI]: 50.4–67.0 months). We observed 166 events of MM relapse, with 47 (92.2%) events in the Ki67 low group and 119 (96.0%) events in the Ki67 high group. The median RFS was 14.0 months (95% CI: 10.5–19.7 months), and the 1-year, 2-year, and 5-year RFS rates were 54.7%, 36.5%, and 12.7%, respectively. Until December 1, 2021, death was recorded in 104 patients (59.4%), with 23 (45.1%) events in the Ki67 low group and 81 (65.3%) events in the Ki67 high group. The median MSS was 42.4 months (95% CI: 35.5–53.3 months), and the 1-year, 2-year, and 5-year MSS were 89.5%, 70.7%, and 40.7%, respectively. Compared with HDI therapy, TMZ-based adjuvant chemotherapy improved RFS in patients with resectable MM (21.0 vs. 9.6 months, P = 0.002, **Figure 2A**). However, there was no significant difference between the two regimens on MSS (45.9 vs. 37.6 months, P = 0.396; **Figure 2B**).

### Prediction of Ki67 Level for Adjuvant Therapy Efficacy

**Figure 3:**
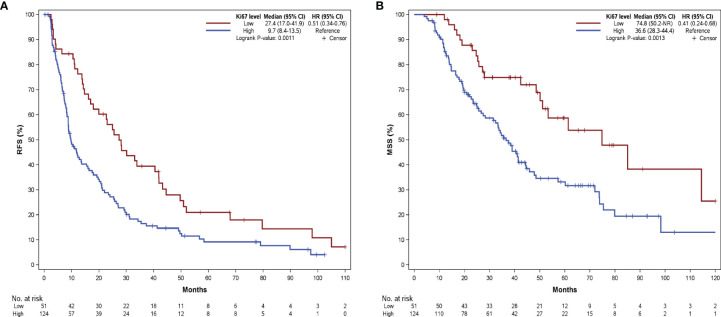
Kaplan-Meier curves of RFS **(A)** and MSS **(B)** according to patients at different Ki67 levels. RFS, relapse-free survival; MSS, melanoma-specific survival.

We performed a multivariate Cox regression taking Ki67 level as the continuous format, and results showed that Ki67 level was an independent factor affecting RFS (HR = 1.029, 95% CI: 1.019–1.038). We then used stratified analysis for subgroup analysis. The clinicopathologic features of the two groups are summarized in **Table 2**. The Kaplan‐Meier survival analysis showed a significant difference between the two Ki67 categories for RFS (P = 0.001, **Figure 3A**) and MSS (P = 0.001, **Figure 3B**). Patients in the Ki67 low group had longer RFS (27.4 vs. 9.7 months) and MSS (74.8 vs. 36.6 months) compared to the Ki67 high group.

**Table 2:** Clinical characteristics of each Ki67 group (<30% and >=30%).

Characteristic	Ki67<30%	Ki67>=30%	P-value
	(n = 51)	(n = 124)	
	No. %	No. %	
**Age**			
Median	62.0	59.0	
Range	31.0-83.0	26.0-86.0	
**Age category**			0.289
<65	32 (62.7)	88 (71.0)	
≥65	19 (37.3)	36 (29.0)	
**Sex**			0.300
Female	29 (56.9)	82 (66.1)	
Male	22 (43.1)	42 (33.9)	
**Primary site**			0.209
Nasal cavity and sinuses	22 (43.1)	42 (33.9)	
Oral cavity	4 (7.8)	13 (10.5)	
Anorectal	6 (11.8)	23 (18.5)	
Genital tract*	13 (25.5)	34 (27.4)	
Urinary tract	4 (7.8)	2 (1.6)	
Esophagus	2 (4.0)	10 (8.1)	
**Mutation status**			0.440
*BRAF*	3 (5.9)	6 (4.8)	
*c-KIT*	1 (2.0)	7 (5.6)	
*NRAS*	2 (3.9)	14 (11.3)	
Wild type	37 (72.5)	79 (63.7)	
Unknown	8 (15.7)	18 (14.5)	
**LDH level**			0.694
≤ULN	46 (90.2)	112 (90.3)	
>ULN	4 (7.8)	7 (5.7)	
Unknown	1 (2.0)	5 (4.0)	
**Lymphatic metastasis**			0.086
N	46 (90.2)	98 (79.0)	
Y	5 (9.8)	26 (21.0)	
**Adjuvant therapy**			0.181
TMZ-based Chemotherapy	25 (49.0)	75 (60.5)	
High dose IFN-α2b	26 (51.0)	49 (39.5)	

*Genital tract included the uterine cervix (2 cases in Ki67<30% group and 4 cases in Ki67>=30% group)_and vulvovaginal primary lesions (11 cases in Ki67<30% group and 30 cases in Ki67>=30% group).

LDH, lactate dehydrogenase; ULN, upper limit of normal; TMZ, temozolomide; IFN, interferon.

**Figure 4:**
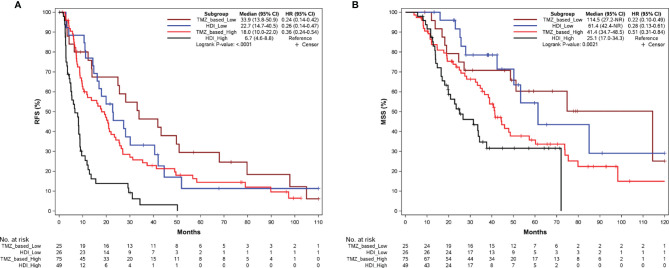
Kaplan-Meier curves of RFS **(A)** and MSS **(B)** according to adjuvant regimens in patients in the Ki67 low (<30%) and Ki67 high (>=30%) group. RFS, relapse-free survival; MSS, melanoma-specific survival; TMZ, temozolomide; HDI, high-dose interferon-a2b.

Then we compared RFS and MSS of patients who received TMZ-based adjuvant chemotherapy or adjuvant HDI therapy at each Ki67 level. In patients with Ki67-low tumors, there was a numerical but not statistically significant difference in RFS with those receiving adjuvant chemotherapy having a median RFS of 33.9 months (95% CI: 13.8–50.9 months, **Figure 4A**) versus 22.7 months (95% CI:14.7–40.5 months, **Figure 4A**) in the HDI group (*P* = 0.329, **Supplementary Figure 1A**). This trend was also noted with regard to median MSS (114.5 vs. 61.4 months, **Figure 4B**) (*P* = 0.967, **Supplementary Figure 1B**). For patients in the Ki67 high group (≥30%), the median RFS after TMZ-based adjuvant chemotherapy and HDI was 18.0 months (95% CI: 10.0–22.0 months, **Figure 4A**) versus 6.7 months (95% CI: 4.6–8.8, **Figure 4A**) *(P* < 0.001, **Supplementary Figure 1C**). In terms of MSS, there was a trend toward improvement with a median of 41.4 months vs 25.1 months (**Figure 4B**) (*P* = 0.067, **Supplementary Figure 1D**).

## Discussion

MM, an aggressive melanoma subtype, is poorly understood in comparison to cutaneous melanoma, owing to its rarity. Lian et al. previously reported the results of two prospective trials of adjuvant treatment in patients with mucosal melanoma, which demonstrated an improvement in RFS with adjuvant TMZ-based chemotherapy compared to observation and/or HDI (6, 7). In our study, 175 patients with resectable MM who underwent adjuvant TMZ-based chemotherapy or HDI therapy were enrolled. Our data show that patients who received TMZ-based adjuvant chemotherapy were able to achieve longer RFS and a slightly higher MSS compared with adjuvant HDI, which is consistent with results of the previous trials. Although adjuvant chemotherapy appears to offer significant benefit, it is not always feasible and can be associated with significant toxicity. As such, we sought to identify a stratification tool to identify patients that may derive the most benefit from adjuvant chemotherapy.

Ki67 is a valuable biomarker of proliferation, and its potential utility in predicting chemotherapy response and long-term outcomes has been explored in various tumors. A series of studies demonstrated that Ki67 was highly correlated with poor prognosis in prostate, lung, serous ovarian and pancreatic cancers (21–26). In breast cancer especially, Ki67 can be used as a determination index for luminal classification and is considered a “therapeutic window” for adjuvant or neoadjuvant therapy (27–31). A Series of studies on melanoma found a correlation between Ki67 expression and patient prognosis, with increased Ki67 expression being associated with an unfavorable prognosis (17, 19, 32, 33). However, its role in adjuvant therapy has not been reported. We performed further exploration and identified that the Ki67 level is an independent factor influencing RFS after adjuvant therapy in patients with resectable MM.

In this study, we observed that for patients with low Ki67 (<30%), the two regimens showed no significant difference in their impact on RFS and MSS. However, for patients with Ki67 ≥ 30%, we observed significantly favorable RFS with TMZ-based adjuvant chemotherapy compared to HDI, which also tended to improve MSS. These results suggest that for resectable MM, patients with Ki67 ≥ 30% can benefit from TMZ-based adjuvant chemotherapy, which may serve as a potential selection indicator for the population benefiting from adjuvant chemotherapy. The criteria for using Ki67 as a stratification basis for adjuvant chemotherapy are widely divergent owing to variations in the malignancy of different tumors and agents used. In breast cancer, Heba et al. reported that patients with a high Ki67 score (>30%) had worse disease-free survival and overall survival after adjuvant tamoxifen therapy and recommended that Ki67 be used as a risk factor in adjuvant treatment decisions (13). However, a study related to adjuvant treatment for breast cancer determined that patients with high Ki67 scores (>30%) could benefit from adjuvant chemotherapy (31). Therefore, we evaluated the role of Ki67 in optimal population selection and stratification for adjuvant therapy in resectable MM to determine the best effect.

The major limitations of our study include three aspects. First, this study was a retrospective study based on a limited sample size, and our findings need to be further validated by multicenter studies with a large sample. Second, there was no observation group in our study; meanwhile, compared with the Ki67 low group, the higher proportion of patients with nodal disease in the Ki67 high group. Therefore, for patients with Ki67 < 30%, the necessity of receiving adjuvant therapy remains to be further validated. Finally, factors associated with an impact on chemotherapy and HDI treatment, such as ulceration, tumor stage and the number of nodal metastases were not included in this study, which may have caused bias in the results and prevented us from verifying whether Ki67 expression can be a precise predictor of therapeutic effect. Ki67 may provide a basis upon which to select patients that are more likely to benefit from adjuvant therapy. Given the potential use of PD-L1 expression as a biomarker for adjuvant immunotherapy in MM and ongoing studies assessing combination immunotherapy + chemotherapy, Ki67 level and PD-L1 expression may serve as a combined biomarker for benefit of adjuvant therapy (10).

To the best of our knowledge, this is the first study to explore a stratification index for adjuvant therapy in patients with resectable MM. We found that patients with Ki67 ≥ 30% can benefit from TMZ-based adjuvant chemotherapy. We have further demonstrated that Ki67 expression as a stratification index based on routine pathology biomarkers can easily distinguish the population benefiting from adjuvant chemotherapy for resectable MM and provide an informative basis for the selection of treatment options.

## Data Availability Statement

The raw data supporting the conclusions of this article will be made available by the authors, without undue reservation.

## Ethics Statement

The studies involving human participants were reviewed and approved by Institutional Review Boards of Peking University Cancer Hospital. The patients/participants provided their written informed consent to participate in this study.

## Author Contributions

LT, XWe, ZL, LS, and JG: research concept and design, data analysis, and interpretation. CL, JD, XB, LM, ZC, CC, BL, BT, YD, XWa, YL, XS, XY, SL, LZ, and YK: provision of study materials or patients. LT and XWe: manuscript writing. All authors edited and approved the final manuscript.

## Funding

This work was supported by grants from the National Natural Science Foundation of China (81972562, 81972566).

## Conflict of Interest

The authors declare that the research was conducted in the absence of any commercial or financial relationships that could be construed as a potential conflict of interest.

## Publisher’s Note

All claims expressed in this article are solely those of the authors and do not necessarily represent those of their affiliated organizations, or those of the publisher, the editors and the reviewers. Any product that may be evaluated in this article, or claim that may be made by its manufacturer, is not guaranteed or endorsed by the publisher.

## Supplementary Material

Supplementary Figure 1 Kaplan-Meier curves of RFS (A) and MSS (B) according to adjuvant regimens in patients in the Ki67 low group (<30%). Kaplan-Meier curves of RFS (C) and MSS (D) according to adjuvant regimens in patients in the Ki67 high group (>=30%). Abbreviations: RFS, relapse-free survival; MSS, melanoma-specific survival; TMZ, temozolomide; HDI, high-dose interferon-a2b.

## Abbreviations

MM, mucosal melanoma; RFS, relapse-free survival; MSS, melanoma-specific survival; TMZ, temozolomide; HDI, high-dose interferon-α2b; PD-1, programmed cell death protein 1; CT, computed tomography; IHC, immunohistochemical.
