# Supplementary figures and images for: Proliferation Marker Ki67 as a Stratification Index of Adjuvant Chemotherapy for Resectable Mucosal Melanoma
Source: Front Oncol. 2022 Jun 30;12:895672. doi: 10.3389/fonc.2022.895672 (PMC9280123; doi:10.3389/fonc.2022.895672)

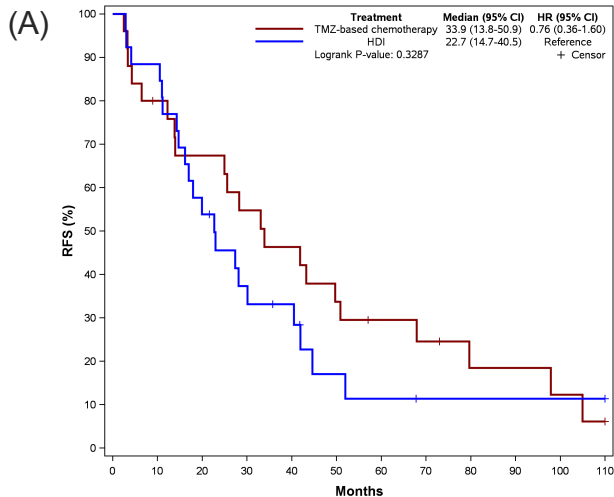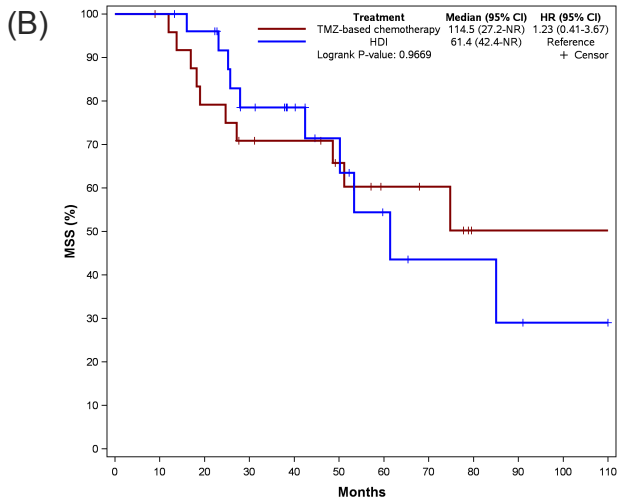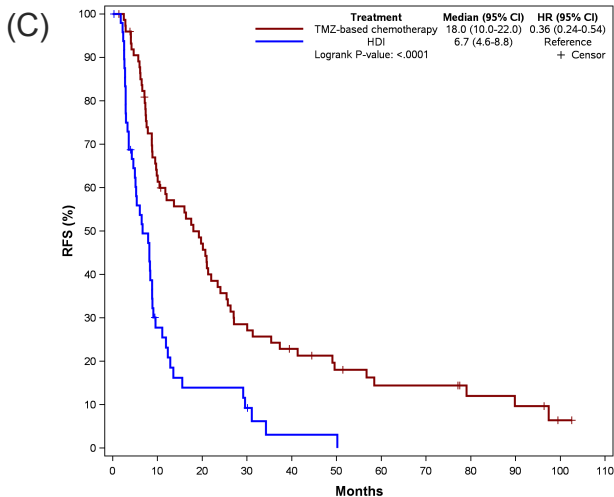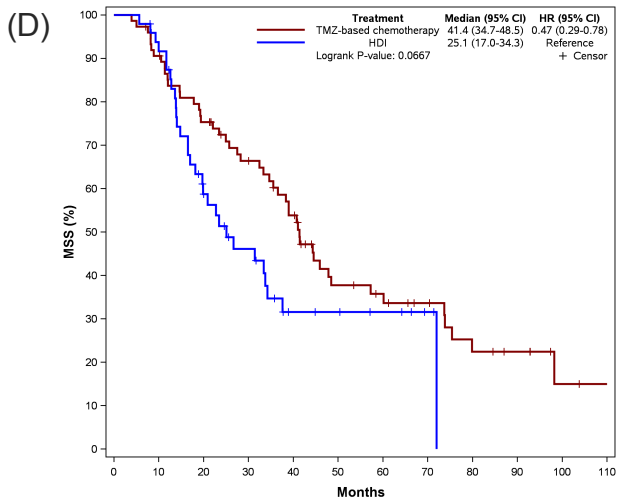

Supplement: Supplementary Figure 1 — Kaplan-Meier curves of RFS (A) and MSS (B) according to adjuvant regimens in patients in the Ki67 low group (<30%). Kaplan-Meier curves of RFS (C) and MSS (D) according to adjuvant regimens in patients in the Ki67 high group (>=30%). Abbreviations: RFS, relapse-free survival; MSS, melanoma-specific survival; TMZ, temozolomide; HDI, high-dose interferon-a2b. [file Image_1.pdf]
